# Psychomotor Delay in a Child with *FGFR3* G380R Pathogenic Mutation Causing Achondroplasia

**DOI:** 10.1055/s-0041-1725070

**Published:** 2021-05-21

**Authors:** Mahmut C. Ergoren, Erdal Eren, Elena Manara, Stefano Paolacci, Pinar Tulay, Sebnem O. Sag, Matteo Bertelli, Gamze Mocan, Sehime Gulsun Temel

**Affiliations:** 1 Department of Medical Genetics, Faculty of Medicine, Near East University, Nicosia, Cyprus; 2 Department of Pediatric Endocrinology, Faculty of Medicine, Bursa Uludağ University, Bursa, Turkey; 3 MAGI’s LAB S.r.l., Rovereto, Italy; 4 Department of Medical Genetics, Faculty of Medicine, Bursa Uludag University, Bursa, Turkey; 5 Department of Histology and Embryology, Faculty of Medicine, Bursa Uludag University, Bursa, Turkey

**Keywords:** achondroplasia, FGFR3 mutations, psychomotor delay

## Abstract

Achondroplasia (ACH) is a hereditary disorder of dwarfism that is caused by the aberrant proliferation and differentiation of chondrocyte growth plates. The common findings of macrocephaly and facial anomalies accompany dwarfism in these patients. *Fibroblast growth factor receptor 3* ( *FGFR3* ) gene mutations are common causes of achondroplasia. The current study presents a case of 2-year-old male child patient presenting with phenotypic characteristics of ACH. The interesting finding of the case is the presence of psychomotor delay that is not very common in these patients. Clinical exome sequencing analyzing 4.813 disease causing genes revealed a de novo c.1138G > A mutation within the *FGFR3* gene. In conclusion, the mutation confirms the clinical diagnosis of ACH, and it seems to be causing the psychomotor delay in this patient.

## Introduction

Achondroplasia (ACH) is characterized by abnormal proliferation and differentiation of the chondrocyte growth plates and endochondral bone growth. It is presented by macrocephaly with distinct facial anomalies, including frontal bossing, depressed nasal bridge, and rhizomelic dwarfism. Further clinical phenotypes include protruding abdomen and bottoms with short hands. Multiple joints, except the elbows, are hyperextensible. 1 Psychomotor delay anomalies can be caused by hydrocephalus, narrow foramen magnum, and spinal canal stenosis. Although it is a rare disorder with a prevalence of 15000 to 40000 live births, ACH is a very common bone dysplasia. 2 Majority of the ACH cases are sporadic. 3 The autosomal dominant inheritance is mainly caused by mutations of *fibroblast growth factor receptor 3* ( *FGFR3* ) gene, which encodes a transmembrane (TM) receptor. *FGFR3* is important in development; therefore, the expression of *FGFR3* has been reported in many tissues, including cartilage, brain, kidneys, and intestines. The mutations within the *FGFR3* gene mainly disturb the chondrocyte proliferation and cartilage development. Thus, it has a direct effect on the phenotype of ACH. 4 The most common mutation was shown to bec.1138G > A (p.G380R). 5 6 Pathogenic mutations, including this one, leads to gain-of-function properties. In this study, we report the genetic diagnosis in a 2-year-old male with clinical indications suggestive of ACH, with the exception of cervicomedullary compression and hydrocephaly.

## Case Report

**Fig. 1:**
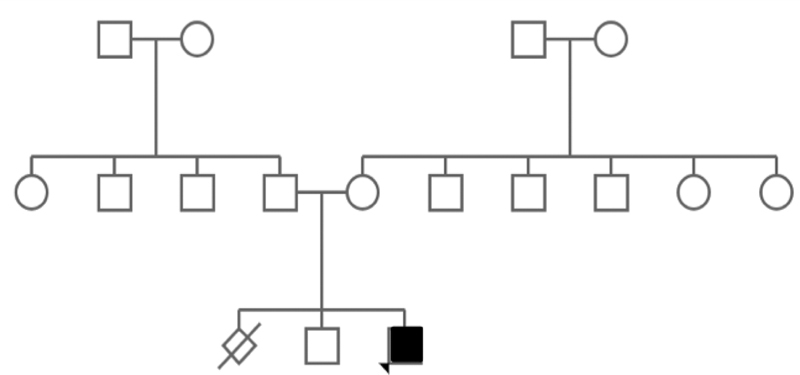
The pedigree of a child with achondroplasia (ACH).

**Fig. 2:**
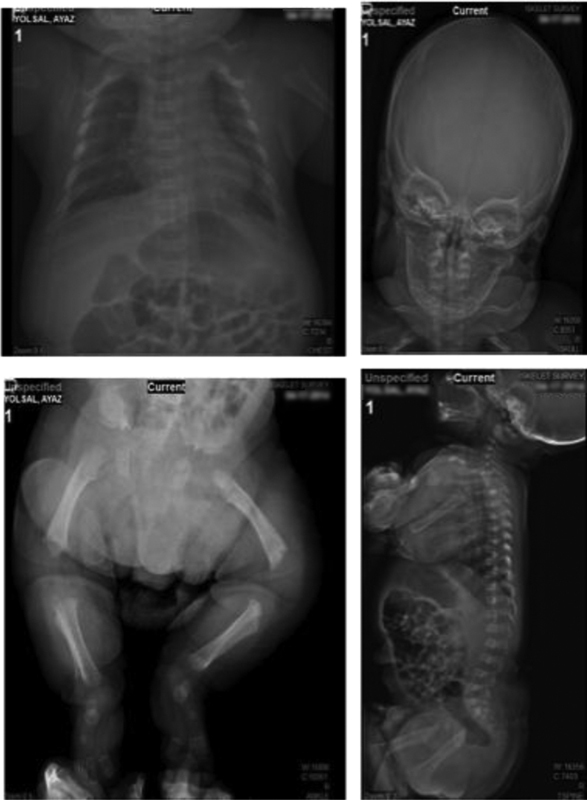
Radiology scan results of a patients' spinal cord, skull, pelvis and lower extremities, respectively.

A 2-year-old male child patient was presented to pediatric endocrinology clinic at the Bursa Uludag University Hospital. He was born via caesarean delivery at 39 weeks of gestation as the second birth of the third pregnancy of a 33-year-old female ( Fig. 1 ). His weight, height, and head circumference were 3340 g, 49 cm, and 35 cm, respectively. The femur shortness of the patient was reported at the 28th week of the gestational period. In the following weeks of the gestation, he was reported to have an increased head circumference. After the delivery, he was admitted to intensive care for 10 days due to respiratory distress. At the 17-month checkup, he weighed 6.5 kg (< 3p), 65 cm (< 3p), and head circumference of 49 cm (50–75p). Physical examination revealed macrocephaly, mild narrow thorax, long body, flatted nose, simian line on the left hand, shortness on the tips of the fingers, and shortness of rhizome in the limbs ( Fig. 2 ). Furthermore, hypotonicity was also reported. Even though, he could control his head, pick up and hold the objects with his thumb and index fingers, turn sideways and crawl backwards; he could not sit without support. His speech was impaired, in such he could only communicate via single words, rather than sentences. Radiologic investigation showed a compression of the interpedicular distance toward the distal end, expansion of the discs and pelvis penetration and short extremities. Cranial MRI showed frontal bossing of the cranium. In the follow-up checkups, he was reported to have delayed neurologic development. At the age of 3.5 years, he weighed 9.8 kg with 74 cm in height, hypotonicity, impairment of walking, distinct hypersensibility, and gibbus deformity. Although these findings were in accordance with ACH phenotype, due to the absence of cervicomedullary compression and hydrocephaly, these findings could also be associated with another skeletal dysplasia. Therefore, genetic testing was offered to the parents of the patient.

## Genetic Diagnosis

Informed consent was obtained from the patient's parents, and whole blood was withdrawn from the patient as well as the parents for genetic analysis. DNA extraction was performed (DNeasy Blood and Tissue Kit, Qiagen, UK) and clinical exome sequencing of 4,813 genes was performed using TruSight One Sequencing Panel (Illumina, UK) on MiSeq platform.

## Results

**Fig. 3:**
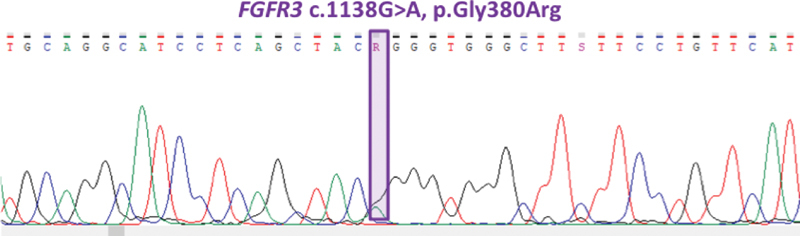
Sequencing result presented a typical achondroplasia (ACH) causing heterozygous *fibroblast growth factor receptor 3* *(FGFR3)* gene G1138A mutation.

Clinical exome sequencing analysis revealed c.1138G > A mutation within the *FGFR3* gene ( Fig. 3 ). This missense mutation led to substitution of glycine with arginine at position 380 (p.G380R) within the FGFR3 protein. The mutation was heterozygous, and it was confirmed by Sanger Sequencing on CEq. 8800 system (Beckman Coulter). The parents' genetic testing showed no mutation within exon 10 of *FGFR3* gene, and therefore it was concluded that this mutation was de novo.

## Discussion

ACH is the most common form of skeletal dysplasia that leads to short-limb dwarfism in humans. 7 The main clinical manifestation is macrocephaly with prominent forehead, flat nasal bridge, and short upper arms and legs. More than 90% of the ACH patients were shown to have a mutation at nucleotide position 1138 on *FGFR3* gene, located on 4p16.3. 5 This gene has shown to have crucial roles in the development of the skeleton. The gene is approximately 15kb and compromises 10 introns and 19 exons. All of these exons encode the TM domain of the gene. Receptor tyrosine kinase FGFR3 has 806 amino acid residues and three domains, including an intracellular region, a TM domain, and an extracellular region. FGF has the ability to bind to acetylated proteins on cells. This attachment stimulates the receptor dimerization and tyrosine kinase transautophosphorylation. 8

In the current study, the genetic diagnosis of a male patient with the clinical manifestation suggestive of ACH, with the exception of cervicomedullary compression and hydrocephaly, is presented. The interesting side of this case is the presence of psychomotor delay, which is not very common in ACH patients. The clinical exome sequencing results revealed *FGFR3* c.1138G > A pathogenic variation, which is a widespread deleterious variation detected in ACH patients. Mutations within the *FGFR3* gene lead to dimerization of cell membrane proteins. Thus, the downregulatory effects may suppress the proliferation and differentiation of cartilage through activating intracellular signaling pathways. 9 This mutation within the *FGFR3* gene is the only finding that could lead to the psychomotor delay in this child with achondroplasia. A scarce number of ACH cases have been reported with neurologic and/or psychomotor delays. 10 11 12 13 However, to our knowledge, none of these have associated the *FGFR3* gene c.1138G > A (p.G380R) pathogenic variation in the ACH patients with psychomotor delay. Thus, this is the first report to associate the detected mutation with the psychomotor deficits. It has been shown previously via computer modeling of this mutation that G380R mutation causes a rotation within the only TM domain dimer of the FGFR3. 14 The contact between two helices was mediated by different amino acids in the mutant dimer compared with the wild type. Previously investigated models using the program CHI revealed the cation‒π interactions between Arg-380 and three aromatic residues (Phe-384 on the same helix; and Tyr-379, Phe-383 on the neighboring helix). 14 The rotation of the TM dimer interface was associated with variations of the kinase activity. The positions of the catalytic domain positions were suggested to be determined by the RTK TM dimer structure. The phosphorylation capacity was also proposed to be directed by the RTK TM dimer structure. Thus, there can be an increase in the phosphorylation, due to the mutation that is caused by the change in the RTK TM dimer structure. Therefore, it has been suggested that the G380R mutation leading to TM dimer rotation causes the catalytic domains to rotate with respect to each other. This rotation of the catalytic domains was proposed to cause higher phosphorylation capacity of the unliganded G380R dimer. 14 Thus, it is a possibility that these structural changes lead to the clinical manifestation of this patient with psychomotor delay, which is not widely observed in the ACH patients.

In conclusion, the detected *FGFR3* c.1138G > A pathogenic variation is the most common cause of ACH. Thus, this mutation detected by sequencing the exons and exon-flanking regions of genes involved in clinical phenotypes is the most likely cause of the neurological manifestation, including the psychomotor delay of the patient with ACH phenotype.

## References

[1] Horton W A Hall J G Hecht J T Achondroplasia Lancet 2007 370(9582):162 172 17630040 10.1016/S0140-6736(07)61090-3

[2] Placone J Hristova K Direct assessment of the effect of the Gly380Arg achondroplasia mutation on FGFR3 dimerization using quantitative imaging FRET PLoS One 2012 7 10 e46678 23056398 10.1371/journal.pone.0046678 PMC3467271

[3] Orioli I M Castilla E E Barbosa-Neto J G The birth prevalence rates for the skeletal dysplasias J Med Genet 1986 23 04 328 332 3746832 10.1136/jmg.23.4.328 PMC1049699

[4] Su N Xu X Li C Generation of Fgfr3 conditional knockout mice Int J Biol Sci 2010 6 04 327 332 20582225 10.7150/ijbs.6.327 PMC2892296

[5] Rousseau F Bonaventure J Legeai-Mallet L Mutations in the gene encoding fibroblast growth factor receptor-3 in achondroplasia Nature 1994 371(6494):252 254 8078586 10.1038/371252a0

[6] Shiang R Thompson L M Zhu Y Z Mutations in the transmembrane domain of FGFR3 cause the most common genetic form of dwarfism, achondroplasia Cell 1994 78 02 335 342 7913883 10.1016/0092-8674(94)90302-6

[7] Kaushal A Haldar R Ambesh P Anesthesia for an achondroplastic individual with coexisting atlantoaxial dislocation Anesth Essays Res 2015 9 03 443 446 26712995 10.4103/0259-1162.158514 PMC4683485

[8] Mohammadi M Dikic I Sorokin A Burgess W H Jaye M Schlessinger J Identification of six novel autophosphorylation sites on fibroblast growth factor receptor 1 and elucidation of their importance in receptor activation and signal transduction Mol Cell Biol 1996 16 03 977 989 8622701 10.1128/mcb.16.3.977 PMC231080

[9] Webster M K Donoghue D J Constitutive activation of fibroblast growth factor receptor 3 by the transmembrane domain point mutation found in achondroplasia EMBO J 1996 15 03 520 527 8599935 PMC449970

[10] Fowler E S Glinski L P Reiser C A Horton V K Pauli R M Biophysical bases for delayed and aberrant motor development in young children with achondroplasia J Dev Behav Pediatr 1997 18 03 143 150 9213228 10.1097/00004703-199706000-00001

[11] Brinkmann G Schlitt H Zorowka P Spranger J Cognitive skills in achondroplasia Am J Med Genet 1993 47 05 800 804 8267016 10.1002/ajmg.1320470540

[12] Ruiz-Garcia M Tovar-Baudin A Del Castillo-Ruiz V Early detection of neurological manifestations in achondroplasia Childs Nerv Syst 1997 13 04 208 213 9202856 10.1007/s003819770001

[13] Hecht J T Thompson N M Weir T Patchell L Horton W A Cognitive and motor skills in achondroplastic infants: neurologic and respiratory correlates Am J Med Genet 1991 41 02 208 211 1785636 10.1002/ajmg.1320410215

[14] He L Horton W Hristova K Physical basis behind achondroplasia, the most common form of human dwarfism J Biol Chem 2010 285 39 30103 30114 20624921 10.1074/jbc.M109.094086 PMC2943285

